# Photochemical activation of carbon dioxide in Mg^+^(CO_2_)(H_2_O)_0,1_

**DOI:** 10.1007/s00214-020-02640-w

**Published:** 2020-07-04

**Authors:** Tobias F. Pascher, Erik Barwa, Christian van der Linde, Martin K. Beyer, Milan Ončák

**Affiliations:** Institut für Ionenphysik und Angewandte Physik, Universität Innsbruck, Technikerstraße 25, 6020 Innsbruck, Austria

**Keywords:** Photoactivation, Carbon dioxide activation, Multi-reference calculations, Spectroscopy

## Abstract

We combine multi-reference ab initio calculations with UV–VIS action spectroscopy to study photochemical activation of CO_2_ on a singly charged magnesium ion, [MgCO_2_(H_2_O)_0,1_]^+^, as a model system for the metal/ligand interactions relevant in CO_2_ photochemistry. For the non-hydrated species, two separated Mg^+^ 3*s*–3*p* bands are observed within 5.0 eV. The low-energy band splits upon hydration with one water molecule. [Mg(CO_2_)]^+^ decomposes highly state-selectively, predominantly via multiphoton processes. Within the low-energy band, CO_2_ is exclusively lost within the excited state manifold. For the high-energy band, an additional pathway becomes accessible: the CO_2_ ligand is activated via a charge transfer, with photochemistry taking place on the CO_2_^–^ moiety eventually leading to a loss of CO after absorption of a second photon. Upon hydration, already excitation into the first and second excited state leads to CO_2_ activation in the excited state minimum; however, CO_2_ predominantly evaporates upon fluorescence or absorption of another photon.

**Electronic supplementary material:**

The online version of this article (10.1007/s00214-020-02640-w) contains supplementary material, which is available to authorized users.

## Introduction

The accurate theoretical description of electronically excited states remains a very difficult but important task for many applications including photocatalysis, [1] light harvesting, [2] photostability, [3, 4] photosensitizers, [5–9] and many more [10]. Already the description of absorption spectra might represent a challenge, e.g., when Rydberg states or charge-transfer excitations are encountered [10]. The complexity is further enhanced outside the Franck–Condon (FC) region, where single-reference methods become insufficient due to state crossings and more demanding multi-reference methods are needed [10–12]. Accurate description of conical intersections (CIs) represents an important task for the understanding of photochemical processes [13–16]. Another layer of difficulty is added in the case of theoretical investigations of metal complexes due to the presence of near-degenerate electronic states and relativistic effects [17].

Catalysis on metals has become a large focus in research due to their importance in, e.g., ammonia and methanol synthesis, [18, 19] but also for photoactivation [1, 20]. Due to its atmospheric relevance, especially activation and transformation of carbon dioxide are addressed in an increasing number of recent studies [21–23]. The direct charge transfer of an electron onto CO_2_ forming an activated bent CO_2_^–^ could be a key step in the activation process. However, the CO_2_^–^ ion itself is metastable [24–27] and has to be hydrated or attached to a metal center to gain stability [28–31]. Only recently, we revealed that hydration of only three water molecules already leads to activation of carbon dioxide forming a CO_2_^–^ ligand on a Mg^2+^ core [32, 33]. The combination of theory and UV–VIS spectroscopy provides a powerful tool for characterization of complicated processes in ionic metal complexes upon excitation [34]. With this approach, we investigated copper formate clusters relevant for carbon dioxide activation on copper centers in the ground state as well as electronically excited states [35–37].

Due to their intriguing charge-transfer chemistry in the ground state, hydrated magnesium ions Mg^+^(H_2_O)_*n*_ have been studied extensively by theory and experiment [38–46]. Photodissociation spectroscopy of hydrated magnesium ions, Mg^+^(H_2_O)_*n*_, provides a suitable model system and benchmark tool for theoretical calculations to investigate the hydrogen production on metal centers [47–51]. Theoretical investigations of Mg^+^ complexes go back to 1991 [52, 53]. Already in 1993, the group of Duncan found partial CO loss in [Mg(CO_2_)]^+^ after excitation within one of the two separated 3*s*–3*p* excitation bands of Mg^+^, pointing toward activation of carbon dioxide within the excited state [54–56]. Whereas the character, vibrational assignment and splitting of these bands was well understood, the photochemical process leading to the activation of the CO_2_ ligand remained unclear due to computational limitations.

Here, we combine investigation of the excited state PES with photodissociation experiments in the gas phase to analyze the photochemical activation of CO_2_ on a Mg^+^ core with and without an additional water molecule.

## Experimental and theoretical methods

The experimental gas-phase action spectra are obtained using FT-ICR mass spectrometry with the cell cooled to the temperature of about 80 K, see Supporting Information for details. For quantum chemical calculations, we used the molecular structures published in Ref. [32] as a starting point. Structures in the ground electronic state are modeled using the Coupled Cluster Singles and Doubles, CCSD/aug-cc-pVDZ, level of theory. For excited state calculations, Equation of Motion CCSD, EOM-CCSD/def2TZVP, and Multi-reference Configuration Interaction, MRCI/def2TZVP, single-point calculations are applied. The def2TZVP basis set is sufficient to describe the orbitals participating in the photochemistry of the system as no Rydberg states are observed among the low-lying excitations. An active space of seven electrons in nine orbitals (7,9) was employed. It includes the valence 3*s* electron of Mg^+^ and six electrons of the CO_2_ ligand which are important for description of the bending coordinate; nine orbitals allow for inclusion of up to six doublet electronic states. Relaxed excited state potential energy surface scans are performed on the Complete Active Space Self-Consistent Field, CASSCF/def2TZVP, level of theory. In comparison with EOM-CCSD, optimization on the CASSCF(7,9)/def2TZVP level of theory yields reasonable structures with similar minima. MRCI(7,9) single-point calculations are performed to include dynamic correlation.

For spectra modeling, we used Franck–Condon simulations [57, 58] as well as the linearized reflection principle within the harmonic approximation [59–61] at the EOM-CCSD/aug-cc-pVDZ level of theory. The Gaussian 16 software was employed for CCSD and EOM-CCSD calculations [62], the Molpro software package for CASSCF and MRCI calculations [63, 64].

## Results and discussion

### UV/VIS spectroscopy

**Fig. 1:**
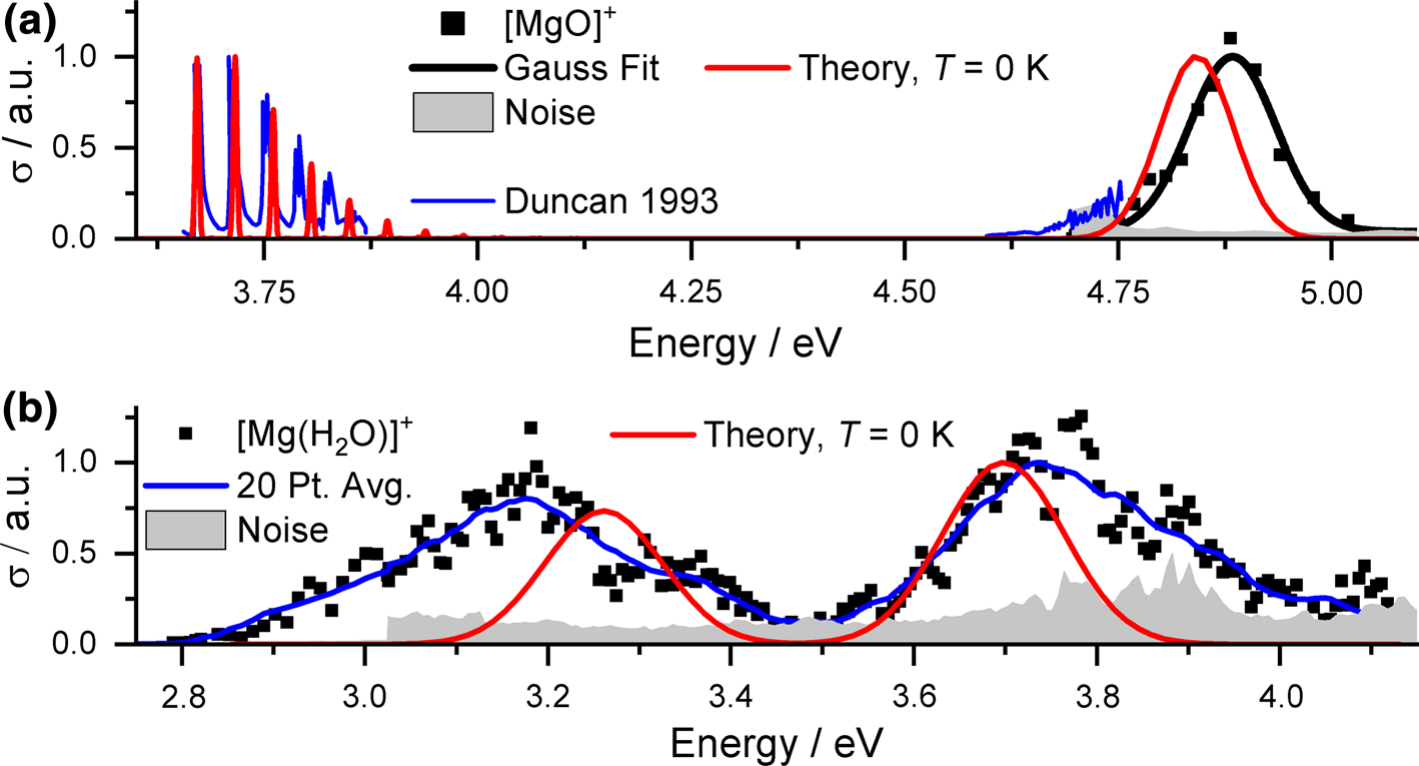
Experimental photodissociation and modeled absorption spectra for (**a**) [Mg(CO_2_)]^+^ and (**b**) [Mg(CO_2_)(H_2_O)]^+^. For modeling, Franck–Condon simulations shifted by 0.046 eV are used for the low-energy band in (**a**), the linearized reflection principle otherwise. The EOM-CCSD/aug-cc-pVDZ//CCSD/aug-cc-pVDZ approach was employed. Data for the blue curve in (**a**) are taken from Ref. [54]

We start our analysis with the spectrum of [Mg(CO_2_)]^+^ from the group of Duncan illustrated in Fig. 1a. At 3.66 eV, a vibrationally resolved absorption band was observed, along with the flank of a second peak at about 4.66 eV [54, 56]. With our tunable OPO system, we have spectral access to the full second absorption, for which we observe a band with a maximum at 4.88 eV, which is fitted very well with a single Gaussian. The resolved vibrational progression in the low-energy band indicates excitation into a bound state while the structureless high-energy band suggests no excited state minimum in the vicinity of the Franck–Condon point in this state. Based on these assumptions, we modeled the spectrum in Fig. 1a. The calculated bands are shifted to slightly lower energies by about 0.05 eV. The vibrational resolution due to the CO_2_ stretching vibration at 360 cm^−1^ in the low-energy band yields a very good agreement between experiment and theory in the population of states even within the harmonic approximation. The width of the high-energy peak is well reproduced within the linearized reflection principle approximation.

Figure 1b shows spectral changes upon hydration in the [Mg(CO_2_)(H_2_O)]^+^ ion. Weak fragment signals corresponding to loss of CO_2_ were obtained in the range from 2.8 to 4.2 eV, where two well-separated bands with similar intensities are observed with maxima around 3.2 and 3.7 eV. Their shape is rather broad, which does not suggest excitation into a bound state near the FC point. Modeling of the spectra based on the linearized reflection principle at *T* = 0 K yields good agreement in excitation energies, with discrepancies smaller than 0.1 eV. However, the experimental width is significantly larger, almost by a factor of two. This points toward thermal effects playing an important role, with a more floppy ground state minimum compared to the case of bare [Mg(CO_2_)]^+^. To improve spectrum modeling, path integral molecular dynamics simulation on the CCSD potential energy surface would be possibly needed, lying beyond the scope of the present study.

### Photochemistry

Decomposition pathways in [Mg(CO_2_)]^+^ highly depend on the investigated band. While for the first band, only CO_2_ loss (reaction (1) in Table 1) was reported by the group of Duncan [54–56], CO loss was observed additionally (reaction (2)) in the flank of the high-energy band. Here, the branching ratio shifted in favor of [MgO]^+^ and reaction (2) toward higher energies [54]. In line with this observation, we only detected the CO loss channel for the high-energy band, suggesting it is the predominant decomposition channel. Presumably due to the poor signal-to-noise ratio in our experiment, we could not detect the competing Mg^+^ fragment reported by Duncan.

**Table 1:** Possible decomposition channels shown in Fig. 1 and Figure S1 with the calculated reaction energy ∆*E*

Reaction	Reactant	Products	*∆E*/eV
(1)	[Mg(CO_2_)]^+^	Mg^+^ + CO_2_	0.65
(1*)		Mg*^+^ + CO_2_	4.97
(2)		[MgO]^+^ + CO	4.52
(3)	[Mg(CO_2_)(H_2_O)]^+^	[Mg(H_2_O)]^+^ + CO_2_	0.48
(3*)		[Mg(H_2_O)]*^+^ + CO_2_	3.98
(4)		[Mg(CO_2_)(OH)]^+^ + H	2.16
(5)		[MgO(H_2_O)]^+^ + CO	2.51
(6)		[Mg(OH)]^+^ + H + CO_2_	3.58
(7)		[Mg(CO_2_)]^+^ + H_2_O	1.10

*Denotes the first excited state. Calculated at the CCSD/aug-cc-pVDZ level along with excitations at the EOM-CCSD/def2-TZVP level of theory

Upon hydration, excitation within the first two absorption bands leads only to CO_2_ molecule loss, reaction (3). This is energetically the most favorable decomposition channel in the ground state, which suggests internal conversion. When the pulse energy is tripled and the number of laser pulses is doubled, [Mg(CO_2_)(OH)]^+^, [MgO(H_2_O)]^+^ and [Mg(OH)]^+^ fragments are observed in smaller amounts starting at about 3.6 eV, see Figure S1. The respective reactions (4–6) would be accessible in the ground state with the available energy after internal conversion. However, the energetically preferred water evaporation is not observed, reaction (7). The observed pulse energy dependence and missing water loss in the experiment thus suggest the involvement of multiphoton processes.

**Fig. 2:**
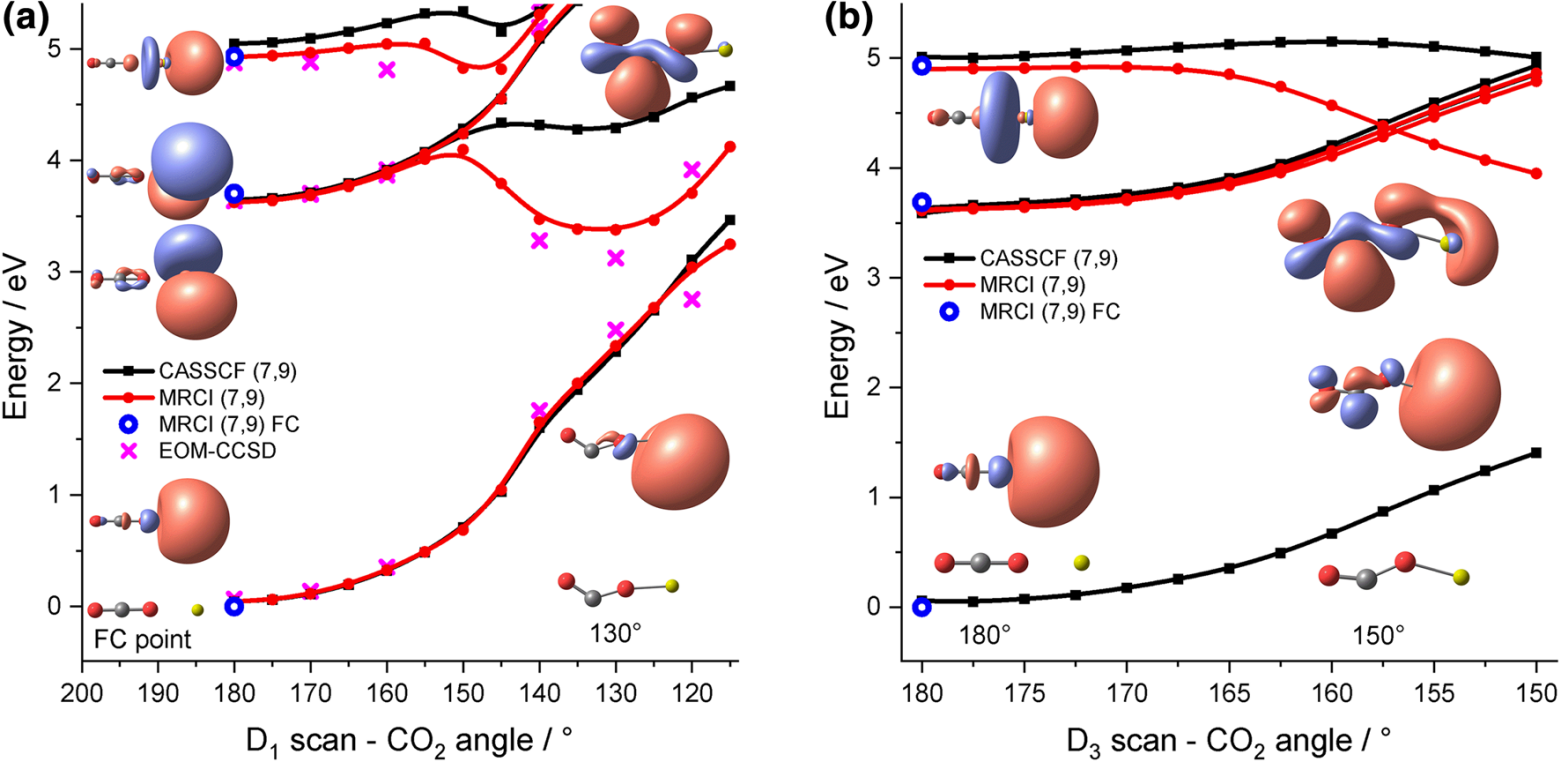
Relaxed PES scan of the CO_2_ angle in [Mg(CO_2_)]^+^ for (**a**) D_1_ and (**b**) D_3_ states at the CASSCF(7,9)/def2-TZVP and MRCI(7,9)//CASSCF(7,9)/def2-TZVP levels. FC point transition energies are given at the MRCI(7,9)/def2-TZVP//CCSD/aug-cc-pVDZ level. EOM-CCSD/def2-TZVP//CASSCF(7,9)/def2-TZVP energies are provided around the minima in (**a**). The structures and the most important singly occupied orbitals according to CASSCF CI vectors are shown for selected points

We start our theoretical photochemical investigation with the linear [Mg(CO_2_)]^+^ complex. In the FC point, the singly occupied molecular orbital in the ground state is the 3*s* orbital of Mg^+^ which is slightly perturbed by the CO_2_ ligand (Fig. 2a). The first excitations correspond to the excitation of the 3*s* electron into 3*p*_*x,y*_ orbitals of Mg^+^ with a vertical excitation energy of 3.70 eV (MRCI(7,9)/def2TZVP//CCSD/aug-cc-pVDZ) in the FC point. These are shifted from three 3*s*–3*p* transitions of bare Mg^+^ at 4.31 eV (MRCI(1,4)/def2TZVP) due to destabilization of the 3*s* orbital upon binding of CO_2_. The excitation at 4.93 eV (MRCI(7,9)/def2TZVP//CCSD/aug-cc-pVDZ) corresponds to excitation into the significantly perturbed 3*p*_*z*_ orbital of Mg^+^, which is collinear with the CO_2_ ligand. This is consistent with previous interpretation and the similar case of [Mg(H_2_O)]^+^ [47, 49, 54–56].

To investigate whether the observed dissociation pathway involving CO loss can be explained by electron transfer from Mg^+^ to the CO_2_ molecule, we performed a relaxed scan along the CO_2_ angle for the D_1_ and D_3_ states on the MRCI(7,9)/def2TZVP//CASSCF(7,9)/def2TZVP level of theory in Fig. 2a, b, respectively.

The D_1_ minimum in the direct vicinity of the FC point lies only 0.08 eV below the D_1_ excitation energy, still with a linear CO_2_ molecule and minimal structural changes. This minimum is well separated with a barrier of about 0.48 eV from an energetically lower-lying D_1_ minimum where CO_2_ is activated with an O–C–O angle of about 130°. Here, the electron is transferred from the Mg^+^ center to the antibonding π* orbital of the CO_2_ ligand. During the charge transfer, the originally degenerate 3*p*_*x,y*_ orbitals split. According to the vibrationally resolved experimental spectrum for D_1_, the D_1_ minimum with bound linear CO_2_ is the target state. However, fluorescence from this minimum can only provide an energy of 0.12 eV, which is insufficient for the CO_2_ loss observed according to reaction (1). Similarly, CO_2_ loss within the excited state via reaction (1*) lies too high in energy, requiring 4.97 eV in total. The ground and the excited state manifold are well separated, and no curve crossing seems to be accessible.

However, a D_1_ minimum can be reached after excitation into the D_3_ state without any barrier (Fig. 2b), which corresponds to a charge-transfer complex between an activated, bent CO_2_^–^ radical anion and Mg^2+^. This observation is consistent with the experimental structureless absorption band suggesting a dissociative shape of the PES around the FC point for excitation into D_3_. Orbital analysis of the relaxed structure in Fig. 2b confirms that the singly occupied 3*p*_*z*_-orbital of Mg^+^ mixes with the antibonding π* orbital of the carbon dioxide ligand upon bending. In the D_1_ minimum, the electron is fully transferred from Mg^+^ to the antibonding π* orbital of the CO_2_ molecule, the C–O bonds are slightly weakened (the one next to Mg from 1.19 to 1.27 Å) while the Mg–O bond is significantly strengthened (from 2.12 to 1.73 Å). However, for dissociation of the CO molecule according to reaction (2), the electron still needs to be transferred to one of the two isoenergetic *p* orbitals of the oxygen ligand in [MgO]^+^. Optimization of a conical intersection toward these states suggests that this process costs about 5.7 eV (EOM-CCSD/def2TZVP), see Figure S2 for the respective interpolation. Thus, the electron transfer along this decomposition channel is not accessible with the energy available upon excitation into D_3_. The dissociation of CO_2_ within the excited state, reaction (1*), is likely inaccessible, given the thermal energy calculated as 0.03 eV at 80 K. Curve crossings into the ground state seem unattainable with the available energy as well.

**Fig. 3:**
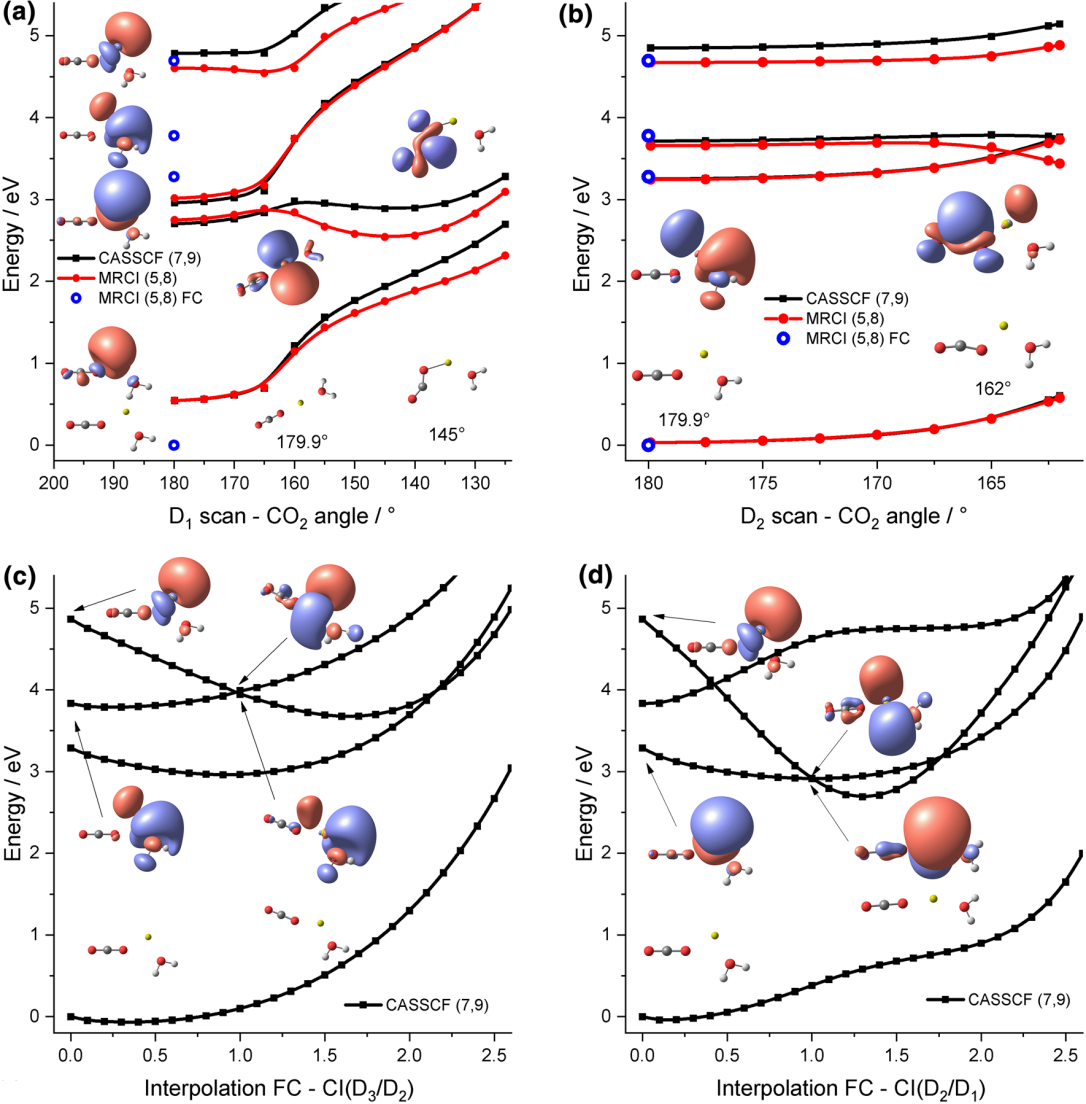
Relaxed PES scan of the CO_2_ angle in [Mg(CO_2_)(H_2_O)]^+^ for (**a**) D_1_ and (**b**) D_2_ at the CASSCF(7,9)/def2-TZVP and MRCI(5,8)//CASSCF(7,9)/def2-TZVP along with the FC point excitation energies at the MRCI(5,8)/def2-TZVP//CCSD/aug-cc-pVDZ level of theory. The structures and the most important singly occupied orbitals according to CASSCF CI vectors are shown for selected structure and states. Interpolation between the FC point and the conical intersection of (**c**) D_3_/D_2_ and (**d**) D_2_/D_1_

In planar [Mg(CO_2_)(H_2_O)]^+^, the weakly bound CO_2_ ligand is oriented end-on on the same side of the Mg^+^ core as the more strongly bound water molecule. The 3*s* and two 3*p* orbitals are perturbed by the ligands (Fig. 3a), only the 3*p* orbital perpendicular to the molecular plane stays unperturbed: The excitations into the D_1_ and D_3_ states are shifted to lower energies of 3.28 and 4.70 eV, respectively, the excitation into the D_2_ state remains almost unshifted at 3.78 eV.

To investigate how the hydration changes the photochemical activation of CO_2_ in the excited state, relaxed CO_2_ angle scans for the D_1_ and D_2_ states are shown in Fig. 3a, b. For D_1_, multiple minima are found again, with an almost linear and a bent CO_2_ molecule (note that another minimum with bent CO_2_ and a very similar energy in D_1_ exists with a flipped CO_2_ bending angle, see Figure S3 for the respective scan). The D_1_ minimum with linear CO_2_ has a dramatically different structure compared to the FC point. After promoting the electron from the 3*s* to the 3*p* orbital of Mg^+^, the ion linearizes analogously to [Mg(H_2_O)_2_]^+^ [47, 49] and the water molecule rotates by 90° to maximize the interaction of the positively charged hydrogen atoms with the electron in the 3*p* orbital of Mg^+^, gaining 0.53 eV of internal energy. Contrary to the case without the water ligand, the bent minimum with an activated CO_2_^–^ ligand is now accessible with the energy available after excitation. CO loss from this minimum would only require 2.51 eV for the hydrated case, reaction (5). However, the charge transfer into the *p* orbital of the oxygen to access this decomposition channel from D_1_ is still very expensive with at least 5.45 eV (EOM-CCSD/def2TZVP), similar to the case without water, see Figure S4 for the respective interpolation. Likewise, decomposition within the excited state manifold is not accessible with the available energy of a single photon, see reaction (3*). Contrary to the case without the water ligand, the gained internal energy after fluorescence from any D_1_ minimum is enough for CO_2_ evaporation, see reaction (3).

After excitation into D_2_, the D_1_/D_2_ conical intersection can be reached barrierlessly, see Fig. 3b. Again, the antibonding π* orbital of the carbon dioxide ligand mixes with the occupied 3*p* orbital from Mg^+^ in the optimized state upon bending. Afterward, access to a similar bent minimum involving a CO_2_^–^ ligand in the D_1_ state can be expected and similar access to decomposition channels with the available energy.

We also investigated the reaction pathways after excitation into the D_3_ state and found pathways for direct internal conversion into D_2_ and D_1_ states (see Fig. 3c, d for interpolations). In both cases, the PES leads monotonically downhill from the FC point to the conical intersections. The orbital analysis of the CIs in Fig. 3c, d shows that for the D_3_/D_2_ CI, the water molecule and CO_2_ rearrange to perturb the 3*p* orbital of Mg^+^ which is occupied in D_2_ while providing more space for the 3*p* orbital populated in D_3_. For the CI into D_1_, the two ligands rearrange to provide space for two 3*p* orbitals perpendicular to the molecular axis. However, the ground state is well separated in all scans. The excitation energy into D_3_ is sufficient to evaporate a CO_2_ molecule from Mg^*+^ via reaction (3*).

**Fig. 4:**
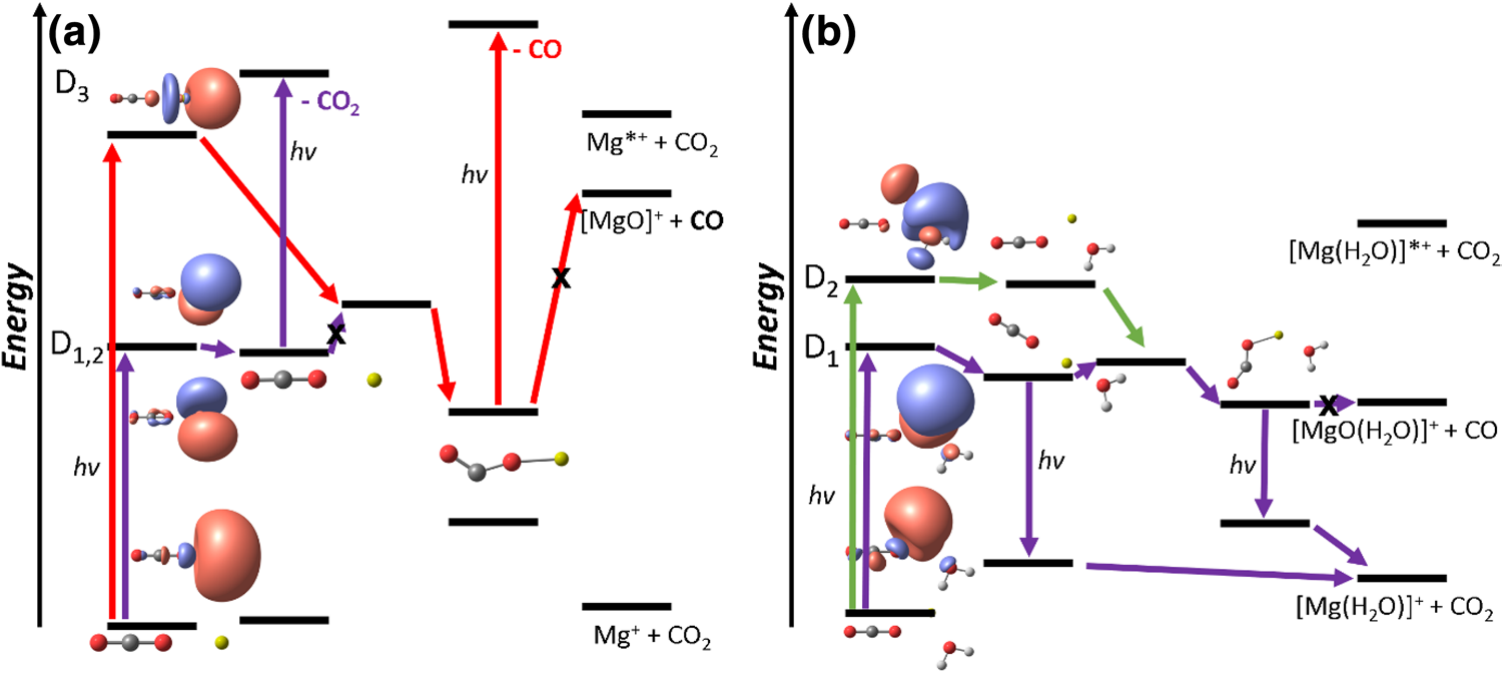
Simplified reaction scheme illustrating the predominantly observed experimental dissociation channels in Fig. 1 for (**a**) [Mg(CO_2_)]^+^ and (**b**) [Mg(CO_2_)(H_2_O)]^+^

The PES scheme in Fig. 4 summarizes our findings. For [Mg(CO_2_)]^+^, excitation into D_1,2_ leads to a bound state where it can only absorb another photon in order to decompose. From the linear D_1_ minimum, several higher lying states are resonantly accessible with an excitation energy of 3.2–4.0 eV (EOM-CCSD/aug-cc-pVTZ). These transitions mostly correspond to excitation of the electron into the empty 3*d* orbitals of Mg^+^. Internal conversion finally leads to CO_2_ loss. Formation of MgO^+^ can happen through an intermediate step after excitation from the ground state into D_3_. Here, an electron can be transferred from Mg*^+^ to the CO_2_ molecule, weakening the C–O bond. However, the observed CO loss is hindered by a significant barrier. Decomposition after internal conversion into the ground state of [Mg(CO_2_)]^+^ can be ruled out as reaction (1) was not observed in the high-energy band in our experiment which should have a significant contribution otherwise. Furthermore, the states are well separated in calculations. Therefore, the CO loss rather takes place photochemically through absorption of an additional photon from the D_1_ minimum involving the CO_2_^–^ unit. With the excitation energy, higher lying states are accessible around an excitation energy of about 4.6–4.9 eV (EOM-CCSD/aug-cc-pVTZ). Contrary to the multiphoton excitation in the D_1_ minimum with a linear CO_2_ molecule, we excite here directly a lone pair electron in the CO_2_^–^ ligand to its out of the bending plane π^*^ orbital while Mg^2+^ plays a minor role. This explains the different photochemical decomposition pathways in the two experimental bands. Fluorescence or multiphoton excitation into 3*p*_z_, which requires about 4.5 eV (EOM-CCSD/aug-cc-pVTZ) in the bent D_1_ minimum, can explain the partially observed CO_2_ loss in the flank of the high energy band previously observed by the group of Duncan [54].

In [Mg(CO_2_)(H_2_O)]^+^, CO_2_ loss can already happen after fluorescing a photon in the linear D_1_ minimum or the D_1_ minima with a bent CO_2_. Upon excitation into D_2_, a CI into D_1_ can be accessed allowing similar decomposition pathways. Direct decomposition via CO loss is hindered again by a barrier. Decomposition after internal conversion into the ground state can be ruled out because water loss (reaction (7)) is not observed in our cooled experiment and the ground state is separated from the excited state manifold. Therefore, the [Mg(CO_2_)(OH)]^+^, [MgO(H_2_O)]^+^ and [Mg(OH)]^+^ fragments observed only with significantly higher laser power and with shifting branching ratios, see Figure S1, occur via multiphoton processes. Excitations into higher lying states are accessible with resonant excitation energies of about 3.6 and 3.5–4.2 eV (EOM-CCSD/aug-cc-pVTZ) in the bent D_1_ minimum of Fig. 3a and its flipped version in Figure S3, respectively.

Furthermore, formation of [Mg(OH)]^+^ as the second most intense channel likely occurs sequentially by absorption of the predominant [Mg(H_2_O)]^+^ fragment as it has an intense absorption band in this range and loses an H atom [47, 49]. Additionally, H loss from the comparable [Mg(H_2_O)_2_]^+^ case happens via a multiphoton-process, in line with our interpretation [47, 49].

## Conclusion

We investigated the photochemistry of [Mg(CO_2_)]^+^ and [Mg(CO_2_)(H_2_O)]^+^ as model systems for the role of metal/ligand interactions in the photochemical activation of CO_2_ by a combination of ab initio calculations and mass spectrometry experiments in the gas phase. The observed decomposition channels are highly state selective. [Mg(CO_2_)]^+^ loses the CO_2_ ligand in the low-energy 3*s*–3*p*_*x,y*_ band via a multiphoton excitation into the Mg 3*d* shell. With the provided energy of the high-energy 3*s*–3*p*_*z*_ band, CO_2_ is activated through a charge transfer from Mg*^+^ in the excited state manifold and forms a bent CO_2_^–^ ligand on a Mg^2+^ center. This leads to predominant CO loss after absorption of an additional photon by the CO_2_^–^ ligand.

Upon hydration with one water molecule, CO_2_ is activated already in the low-energy 3*s*–3*p* band. The rearrangement in the D_1_ minima provides enough internal energy for CO_2_ loss through fluorescence in addition to the previous multiphoton process. The [Mg(CO_2_)(OH)]^+^, [MgO(H_2_O)]^+^ and [Mg(OH)]^+^ fragments in the D_2_ band arise from an additional excitation in D_1_ minima in combination with sequential fragmentation of [Mg(H_2_O)]^+^ to [Mg(OH)]^+^. For the third 3*s*–3*p* transitions, we predict direct funneling into the first excited state, providing enough energy to directly evaporate a CO_2_ molecule on the excited state PES.

## Electronic supplementary material

Below is the link to the electronic supplementary material.

Supplementary material 1 (PDF 752 kb)
