# Supplementary material for: Photochemical activation of carbon dioxide in Mg+(CO2)(H2O)0,1
Source: Theor Chem Acc. 2020 Jul 4;139(8):127. doi: 10.1007/s00214-020-02640-w (PMC7335376; doi:10.1007/s00214-020-02640-w)
Supplement: Supplementary file 1 — Supplementary material 1 (PDF 752 kb) [file 214_2020_2640_MOESM1_ESM.pdf]

Supporting Information:

Photochemical Activation of Carbon Dioxide in $Mg^+(CO_2)(H_2O)_{0,1}$

Tobias F. Pascher, Erik Barwa, Christian van der Linde, Martin K. Beyer*, Milan Ončák*

Institut für Ionenphysik und Angewandte Physik, Universität Innsbruck, Technikerstraße 25, 6020 Innsbruck, Austria

* E-mail: martin.beyer@uibk.ac.at; milan.oncak@uibk.ac.at

Experimental Methods

The experiments were performed on a modified 4.7 T FT-ICR Bruker/Spektrospin CMS47X mass spectrometer[1–3] equipped with a Bruker infinity cell[4] and an external laser vaporization source.[5–7] The ions are irradiated within the ICR cell cooled to liquid nitrogen temperature ($T \approx 80$ K) [8] under ultra-high vacuum conditions (~10^{-10} mbar) by UV-VIS photons from a tunable Ekspla NT342B OPO laser system calibrated with an Ocean Optics USB2000+ spectrometer. The experimental procedure involving Mg is described in more detail in Ref. [9]. The cross section is calculated as described previously within Ref. [10, 11] in arbitrary units. A correction factor has been applied at 410 nm to account for the changing laser beam alignment and beam profile upon switching from the signal to the sum frequency generation stage of the OPO system.

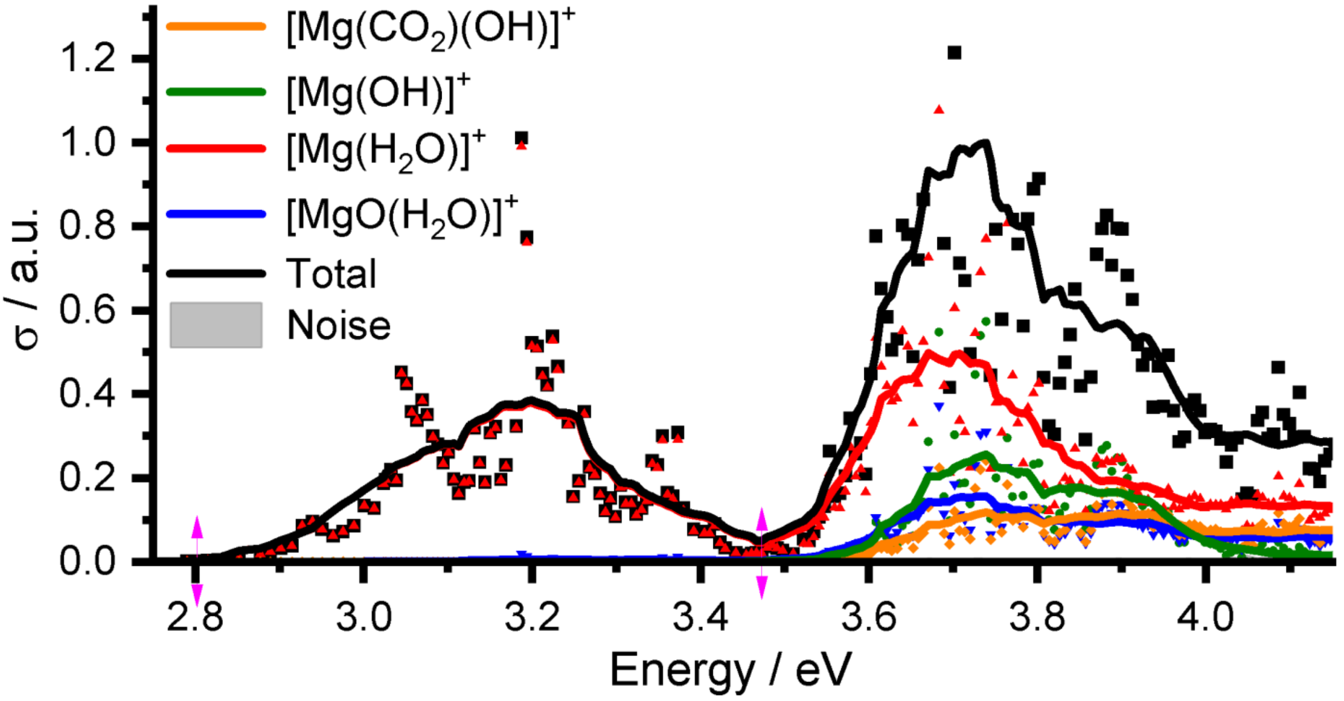

Figure S1. Photodissociation spectrum of $[Mg(CO_2)(H_2O)]^+$ along with decomposition into fragmentation channels using a running average of 20. Here, clusters were excited by a doubled amount of pulses (20) along with an ~2.5–3 times higher laser power in comparison to Figure 1b.

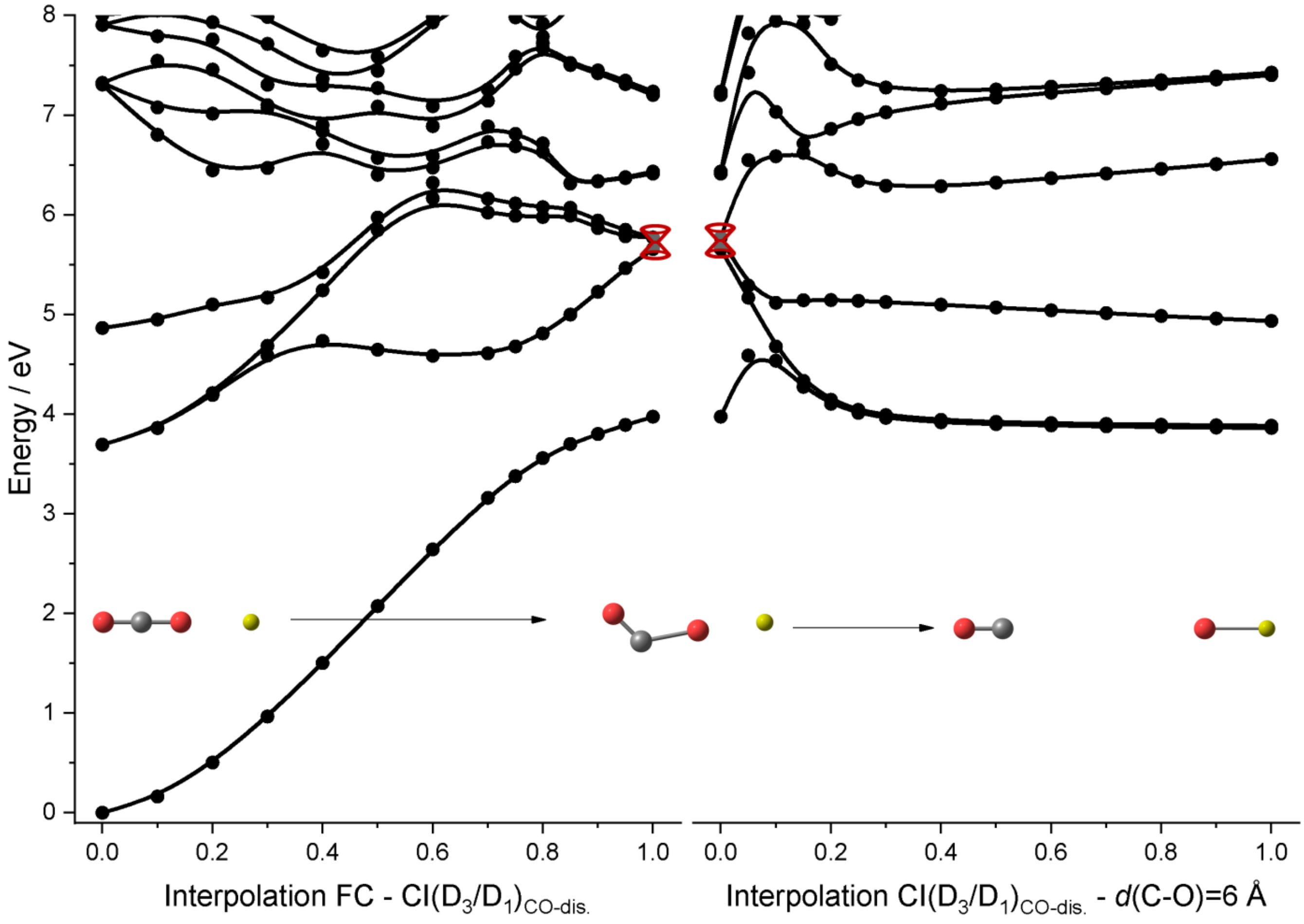

Figure S2. Interpolation for $[Mg(CO_2)]^+$ at the EOM-CCSD/def2TZVP level of theory between the FC point and the CI ($D_1/D_2/D_3$) allowing access to the charge transfer towards to the ground states of $[MgO]^+$ after the CO dissociation, left, and interpolation between the CI towards the dissociation of CO with a fixed $[MgO]^+$-CO bond distance *d*(C-O) of 6 Å. The FC point and $[MgO]^+$-CO structure are obtained at the CCSD/aug-cc-pVDZ level of theory while the CI are obtained employing EOM-CCSD/def2TZVP as optimization on the CASSCF level of theory would require too large active space. For the CI optimization at the EOM-CCSD level of theory, the optimization starts to oscillate around the CI. Therefore, a reduced convergence criterion of $<2.0*10^{-8}$ Hartree in predicted energy change was used. The obtained points are b-splined to guide the eye.

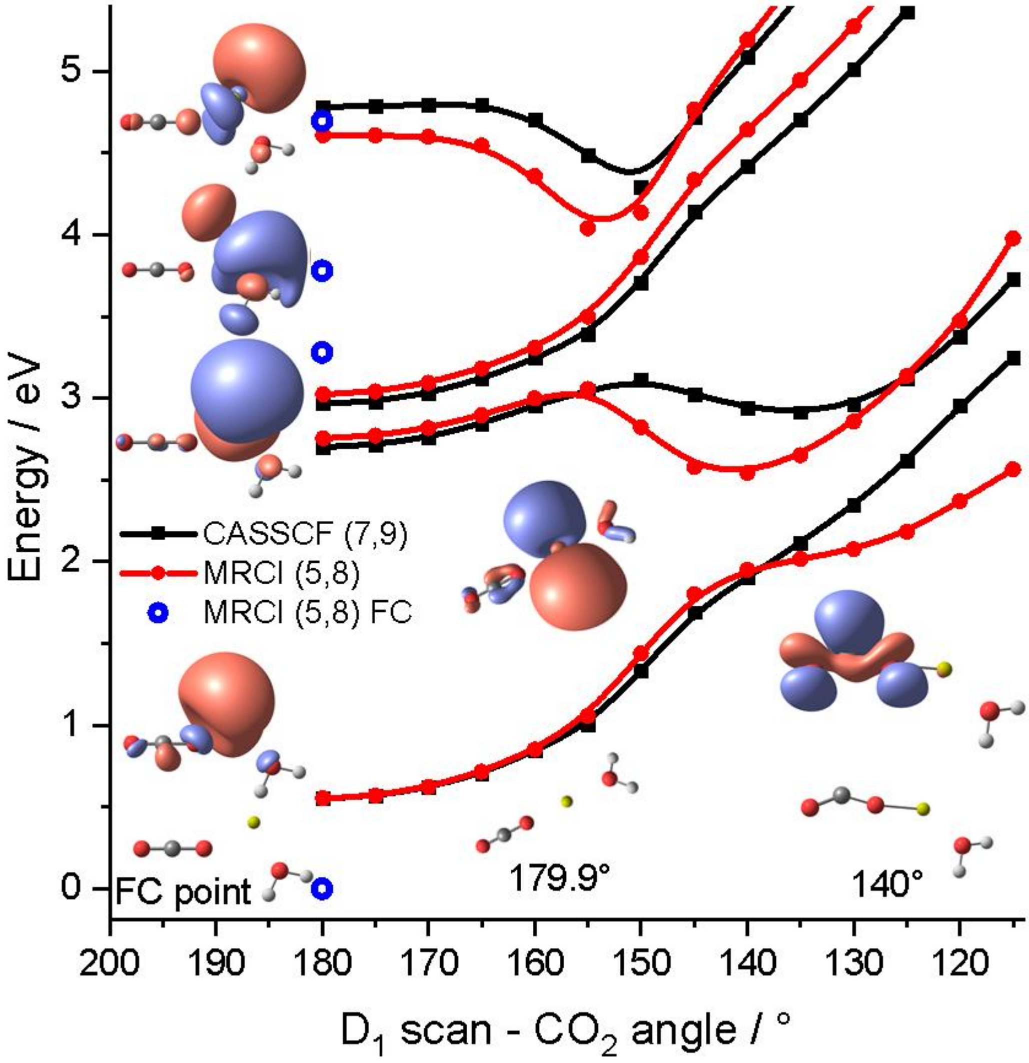

Figure S3. Relaxed PES scan of the CO_2 angle in $[Mg(CO_2)(H_2O)]^+$ for D_1 at the CASSCF(7,9)/def2-TZVP and MRCI(5,8)//CASSCF(7,9)/def2-TZVP. FC point excitation energies are given at the MRCI(5,8)/def2-TZVP//CCSD/aug-cc-pVDZ level of theory. The structures and the most important orbitals according to CI coefficients of the ground state and respective optimized state are shown for selected points.

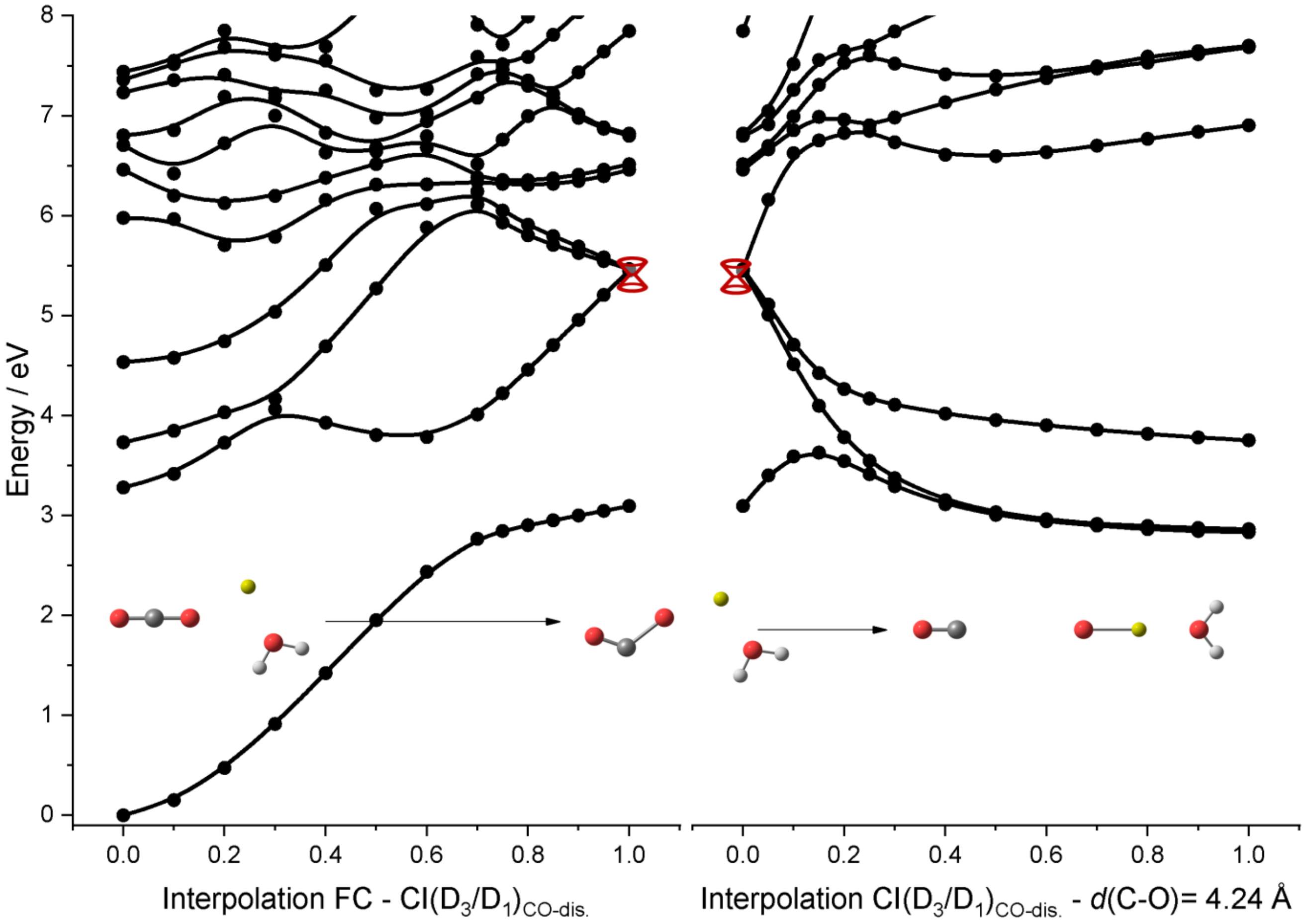

Figure S4. Interpolation for $[Mg(CO_2)(H_2O)]^+$ at the EOM-CCSD/def2TZVP level of theory between the FC point and the CI($D_3/D_2/D_1$) allowing access to the charge transfer towards the ground states of $[Mg(H_2O)O]^+$ after the CO dissociation, left, and interpolation between the CI towards the dissociation of CO at a $[Mg(H_2O)O]^+$-CO bond distance *d*(C-O) of 4.24 Å. The FC point and $[Mg(H_2O)O]^+$-CO structure are obtained at the CCSD/aug-cc-pVDZ level of theory while the CI is obtained on the EOM-CCSD/def2TZVP as optimization on the CASSCF level of theory would too large active space. For the CI optimization at the EOM-CCSD level of theory, the optimization starts to oscillate around the CI. Therefore, a reduced convergence criterion of $<1.0*10^{-7}$ Hartree in predicted energy change was used. The obtained points are b-splined to guide the eye. Note that a similar CI exists with a flipped CO_2 bending angle. However, it lies about 0.15 eV higher in energy. (Optimized via steepest decent at a reduced convergence criterion of $<5.0*10^{-9}$ Hartree.)

Literature

1. Allemann M, Kellerhals H, Wanczek KP (1983) High Magnetic-Field Fourier-Transform Ion-Cyclotron Resonance Spectroscopy. Int J Mass Spectrom Ion Process 46(January): 139–142. doi: 10.1016/0020-7381(83)80073-4

2. Schindler T, Berg C, Niedner-Schatteburg G, Bondybey VE (1995) Reactions of water clusters $H^+(H_2O)_n$, n=3-75, with diethyl ether. Chem Phys 201(2-3): 491–496. doi: 10.1016/0301-0104(95)00288-X
3. Marshall AG, Hendrickson CL, Jackson GS (1998) Fourier transform ion cyclotron resonance mass spectrometry: A primer. Mass Spectrom Rev 17(1): 1–35. doi: 10.1002/(SICI)1098-2787(1998)17:1<1:AID-MAS1>3.0.CO;2-K
4. Caravatti P, Allemann M (1991) The Infinity Cell: A New Trapped-Ion Cell with Radiofrequency Covered Trapping Electrodes for Fourier Transform Ion Cyclotron Resonance Mass Spectrometry. Org Mass Spectrom 26(5): 514–518. doi: 10.1002/oms.1210260527
5. Bondybey VE, English JH (1981) Laser Induced Fluorescence of Metal Clusters Produced by Laser Vaporization: Gas Phase Spectrum of Pb_2. J Chem Phys 74(12): 6978–6979. doi: 10.1063/1.441064
6. Dietz TG, Duncan MA, Powers DE, Smalley RE (1981) Laser Production of Supersonic Metal Cluster Beams. J Chem Phys 74(11): 6511–6512. doi: 10.1063/1.440991
7. Berg C, Schindler T, Niedner-Schatteburg G, Bondybey VE (1995) Reactions of Simple Hydrocarbons with Nb_n^+: Chemisorption and Physisorption on Ionized Niobium Clusters. J Chem Phys 102(12): 4870–4884. doi: 10.1063/1.469535
8. Balaj OP, Berg CB, Reitmeier SJ, Bondybey VE, Beyer MK (2009) A novel design of a temperature-controlled FT-ICR cell for low-temperature black-body infrared radiative dissociation (BIRD) studies of hydrated ions. Int J Mass Spectrom 279(1): 5–9. doi: 10.1016/j.ijms.2008.09.001
9. Barwa E, Pascher TF, Ončák M, van der Linde C, Beyer MK (2020) Carbon Dioxide Activation at Metal Centers: Evolution of Charge Transfer from $Mg^{\cdot+}$ to CO_2 in $[MgCO_2(H_2O)_n^{\cdot+}$, n=0-8. Angew Chem Int Ed 59: 7467-7471. doi: 10.1002/anie.202001292
10. Ončák M, Taxer T, Barwa E, van der Linde C, Beyer MK (2018) Photochemistry and Spectroscopy of Small Hydrated Magnesium Clusters $Mg^+(H_2O)_n$, n = 1-5. J Chem Phys 149(4): 44309. doi: 10.1063/1.5037401
11. Herburger A, Barwa E, Ončák M, Heller J, van der Linde C, Neumark DM, Beyer MK (2019) Probing the Structural Evolution of the Hydrated Electron in Water Cluster Anions $(H_2O)_n^-$, $n \leq 200$, by Electronic Absorption Spectroscopy. J Am Chem Soc 141(45): 18000–18003. doi: 10.1021/jacs.9b10347

Cartesian coordinates (in Å) and electronic energies (in Hartree) including zero-point energy as optimized at the noted level of theory

CCSD/aug-cc-pVDZ

CO
E=-113.056365
C 0.000000 0.000000 -0.070249
O 0.000000 0.000000 1.070249

CO2
E=-188.152791
O 0.000000 0.000000 -1.170558
C 0.000000 0.000000 0.000000
O 0.000000 0.000000 1.170558

H2O
E=-76.247185
O 0.000000 -0.000000 0.120253
H 0.000000 0.760867 -0.471862
H -0.000000 -0.760867 -0.471862

H
E=-0.499334
H 0.000000 0.000000 2.142747

Mg(CO2)+
E=-387.541714
C 3.239614 0.000000 0.000000
O 4.388057 0.000000 0.000000
O 2.047728 0.000000 0.000000
Mg -0.075399 0.000000 0.000000

Mg+
E=-199.364918
Mg 0.000000 0.000000 2.142747

Mg(CO2)(H2O)+
E=-463.829436
C 1.745228 -0.145243 0.000000
O 0.556017 -0.092720 0.000000
O 2.894736 -0.205902 0.000000
Mg -1.325799 1.057718 0.000000
O -2.247589 -0.807593 0.000000
H -1.825338 -1.678605 0.000000
H -3.202425 -0.968014 0.000000

Mg(CO2)(OH)+
E=-463.250563
C 1.949039 -0.001799 -0.001799
O 3.092293 -0.038139 -0.038139
O 0.755518 0.037088 0.037088
Mg -1.238631 0.070164 0.070164
O -2.978362 -0.012812 -0.012812
H -3.898922 0.161498 0.161498

Mg(H2O)+
E=-275.659083
Mg 1.000519 0.000000 0.000000
O -1.075283 -0.000000 -0.000000
H -1.660567 0.773275 -0.000000
H -1.660567 -0.773275 0.000000

MgO+
E=-274.319201
Mg 0.000000 0.000000 2.295695
O 0.000000 0.000000 0.547052

MgO(H2O)+
E=-350.680865
O -2.068806 0.000000 0.000000
Mg -0.216880 0.000000 0.000000
O 1.784850 -0.000000 -0.000000
H 2.370555 -0.773613 -0.000000
H 2.370555 0.773613 0.000000

Mg(OH)+
E=-275.045620
Mg 0.008291 0.008291 0.759400
O -0.047845 -0.047845 -0.984923
H 0.289554 0.289554 -1.814125

MgO+ – CO fixed *d*(C-O) = 6 Å
o 0.000000 0.000000 -5.049550
c 0.000000 0.000000 -3.909885
o 0.000000 0.000000 2.090115
mg 0.000000 0.000000 3.934647

Mg(H2O)O+ – CO *d*(C-O) = 4.24 Å
O -0.000000 -0.000000 0.198267
Mg -0.000000 -0.000000 2.049848
O -0.000000 -0.000000 4.053108
H -0.000000 0.773671 4.638671
H -0.000000 -0.773671 4.638671
C 0.000000 0.000000 -4.037703
O 0.000000 0.000000 -5.177570

EOM-CCSD/def2TZVP

Mg(CO2)+ CI(D3/D2/D1) Figure S2

o	-1.097598	-2.018563	0.0
c	-1.249987	-0.894514	0.0
o	0.0	0.231682	0.0
mg	1.356726	1.638511	0.0

Mg(CO2)(H2O)+ CI(D3/D2/D1) Figure S4

c	1.538708	1.253379	0.0
o	0.0	0.689341	0.0
mg	-1.514376	-0.590922	0.0
o	-0.58022	-2.389213	0.0
o	1.770666	2.368348	0.0
h	0.381304	-2.500387	0.0
h	-0.964611	-3.276624	0.0

CASSCF(7,9)/def2TZVP

Mg(CO2)(H2O)+ CI(D3/D2) Figure 3c
C -0.3512288897 1.9440763214 0.0000001505
O -0.0852915628 0.7936905344 0.0000033605
O -0.5570689901 3.0451397090 -0.0000028583
Mg -0.3476283942 -1.1393470956 0.0000011078
O 1.2819455510 -2.2466720313 -0.0000003052
H 2.1755047788 -1.9293871093 0.0000013618
H 1.2939355071 -3.1945593285 -0.0000028171

Mg(CO2)(H2O)+ CI(D2/D1) Figure 3d
C 0.0000009726 0.0519723928 -1.9556227537
O -0.0000023828 -0.0289248408 -0.7926382396
O 0.0000007925 0.1320501469 -3.0757475066
Mg 0.0000002670 -0.3044044325 1.1715547882
O 0.0000003985 0.2669551648 3.0986782137
H -0.0000019120 1.1581458720 3.4217322133
H 0.0000027995 -0.3116234251 3.8497367851

Mg(CO2)+ D1 bent CO2 minimum in MRCI Figure 2a
α(O-C-O) fixed at 130°
C 0.0000000000 0.4013635838 -1.1020224189
O 0.0000000000 0.0400410212 0.1129374492
Mg 0.0000000000 -0.0642100489 1.8388556841
O 0.0000000000 -0.2438082104 -2.0790734374

Mg(CO2)+ D1 linear CO2 minimum
C 0.0000000000 0.0000000000 -1.1239248668
O 0.0000000000 0.0000000000 0.0465125852
Mg 0.0000000000 0.0000000000 2.0021473543
O 0.0000000000 0.0000000000 -2.2442656179

Mg(CO2)+ D1 nearly linear CO2 minimum Figure 2a
α(O-C-O) fixed at 180.0°
C 0.0000000000 -0.0048068356 -1.1233020098
O 0.0000000000 -0.0242887884 0.0470666628
Mg 0.0000000000 0.0092552767 2.0008722778
O 0.0000000000 0.0138374841 -2.2433502904

Mg(H2O)(CO2)+ D1 bent CO2 minimum in MRCI Figure 3a α(O-C-O) fixed at 145°
C 0.0000000000 -0.2070744362 1.3728200713
O 0.0000000000 -0.7392045991 2.3995885726
O 0.0000000000 0.8901167136 0.7991193526
Mg 0.0000000000 0.6921901929 -1.0706612161
O 0.0000000000 -0.8786189407 -2.2758256406
H 0.0000000000 -1.7759891178 -1.9667046448
H 0.0000000000 -0.8964308553 -3.2241637021

Mg(H2O)(CO2)+ D1 flipped bent minimum in MRCI Figure S3 α(O-C-O) fixed at 140°
C -0.0000661298 -0.2324926511 1.8919515546
O 0.0004589410 0.4855779785 2.8113005427
O -0.0010051418 -0.1885937303 0.6397891102
Mg 0.0004456330 -0.4816576240 -1.0967067982
O -0.0000817767 0.5066280497 -2.7940596780
H -0.0006991542 1.4566449084 -2.8460113922
H 0.0007095102 0.1722647312 -3.6830049911

Mg(H2O)(CO2)+ near linear CO2 D1 minimum Figure S3
α(O-C-O) fixed at 179.9°
C 0.0010446158 0.0012298717 1.9765128835
O 0.0015783582 0.0043211595 3.0990943401
O -0.0015414280 -0.0021104007 0.8102167471
Mg -0.0026022273 -0.0075921471 -1.1700032680
O 0.0024190246 0.0074069639 -3.1494510492
H -0.7645696054 0.0085416261 -3.7052779858
H 0.7758862002 0.0072106554 -3.6962255845
